# Masking our emotions: Emotion recognition and perceived intensity differ by race and use of medical masks

**DOI:** 10.1371/journal.pone.0284108

**Published:** 2023-06-07

**Authors:** Ashley Y. Li, Disha P. Rawal, Vanessa V. Chen, Nathan Hostetler, Shannon A. H. Compton, Emma K. Stewart, Mary B. Ritchie, Derek G. V. Mitchell

**Affiliations:** 1 Brain and Mind Institute, Western Interdisciplinary Research Building, Room 3190, University of Western Ontario, London, Ontario, Canada; 2 Graduate Program in Neuroscience, Schulich School of Medicine and Dentistry, University of Western Ontario, London, Ontario, Canada; 3 Department of Psychology, Faculty of Social Science, University of Western Ontario, London, Ontario, Canada; 4 Department of Psychiatry, Schulich School of Medicine and Dentistry, University of Western Ontario, London, Ontario, Canada; 5 Department of Anatomy and Cell Biology, Schulich School of Medicine and Dentistry, University of Western Ontario, London, Ontario, Canada; Vels Institute of Science, Technology and Advanced Studies (VISTAS), MALAYSIA

## Abstract

Although medical masks have played a key role in decreasing the transmission of communicable disease, they simultaneously reduce the availability of nonverbal cues fundamental to social interaction. In the present study, we determined the collective impact of medical masks on emotional expression recognition and perceived intensity as a function of actor race. Participants completed an emotional expression recognition task involving stimuli with or without medical masks. Across six basic emotional facial expressions, medical masks were associated with significantly more emotional expression recognition errors. Overall, the effects associated with race varied depending on the emotion and appearance of masks. Whereas recognition accuracy was higher for White relative to Black actors for anger and sadness, the opposite pattern was observed for disgust. Medical mask-wearing exacerbated actor-race related recognition differences for anger and surprise, but attenuated these differences for fear. Emotional expression intensity ratings were significantly reduced for all emotions except fear, where masks were associated with increased perceived intensity. Masks further increased already higher intensity ratings for anger in Black versus White actors. In contrast, masks eliminated the tendency to give higher intensity ratings for Black versus White sad and happy facial expressions. Overall, our results suggest that the interaction between actor race and mask wearing status with respect to emotional expression judgements is complex, varying by emotion in both direction and degree. We consider the implications of these results particularly in the context of emotionally charged social contexts, such as in conflict, healthcare, and policing.

## Introduction

Facial expressions serve an important communicatory function, allowing us to signal affiliation, the presence of threats or distress, and otherwise modulate behaviours in others in a variety of contexts. The COVID-19 pandemic led many governments to recommend or order mask mandates in 2020. Although medical mask practices and protocols continue to evolve and vary by setting, community and country, they remain an important part of personal protection in hospitals, and are likely to remain a cost-effective measure in response to future COVID-19 variants, waning immunity, or other airborne viruses [1]. Identifying any potential collateral effects of mask-wearing therefore remains important. The potential impact of masks on social interactions is one area of concern. For example, there is increasing evidence that mask-wearing disrupts our ability to process and interpret emotion in facial expressions [e.g., 2–6], and may adversely impact social closeness, bonding, and other factors related to healthy social interactions [7, 8]. In consideration of this evidence, there is a need to determine how masks may interact with other factors to influence perception of emotions and social interactions. Considering that racial disparities exist in police encounters [9], one potentially important factor is the race of the mask wearer. For example, Black/Brown adults are five times more likely than White adults to report being unfairly stopped by police due to their race or ethnicity [10], and unarmed Black individuals are far more likely to be shot than unarmed White individuals [11]. Studies suggest Black/Brown individuals are stereotyped as aggressive and more threatening than White counterparts [12–14], even those who are 5 years old [15] or elderly [16]. There is also evidence that racial biases exist in the perception of pain, which may have implications for care in healthcare settings, where masks are frequently worn [17]. Taken together, these findings suggest that both mask-wearing and racial and ethnic biases influence perception of threat and other socially relevant expressions. Although it is presently unclear how medical masks interact with race to influence emotion perception, racialized groups have expressed concerns in this regard; in a recent survey, Black and Asian participants reported concerns that wearing masks may increase the tendency to be racially stereotyped as threatening by the general public and police, and that such stereotypes may impact their safety [18].

### Importance of facial expressions for social behaviour

Facial expressions play a key role in modulating the behaviour of others. Anger is of particular importance to social regulation, which involves a dissociable biological response profile to distress [19, 20]. Anger signals disapproval, particularly when social rules or expectations are violated, and tends to result in the cessation or modification of ongoing behaviours [21–23]. Decoding anger in others facilitates the expression of gestures of appeasement, helping alleviate problematic exchanges [24]. Abnormalities in the ability to recognize angry facial expressions are associated with more socially inappropriate behaviours, and an increased risk for aggression in some neurological populations [23, 25]. It should be noted that increased sensitivity to emotions may also be problematic and have been related to conditions that feature maladaptive social behaviours. For example, borderline personality disorder may be associated with enhanced detection of emotional expressions [26], particularly anger in certain contexts [27]. In addition, generalized anxiety disorder is associated with detection of emotional expressions at lower intensities than health controls [28].

Other categories of emotion play a distinct, but critical communicatory role. Distress cues, such as fear and sadness, act as reinforcers, leading associated objects or actions to be experienced as aversive, and less likely to be initiated in the future [22, 29]. In line with this, interpreting distress cues in others has been linked to the interruption of aggression, and the initiation of prosocial behaviours [30, 31]. In contrast, difficulties in recognizing these cues are associated with reduced prosocial behaviour [32], and are especially prominent in populations that feature high rates of antisocial behaviours [33].

### Facial expression processing and race

Considerable research supports the idea that racial bias plays a significant role in the interpretation of emotions in others, and this is particularly true for judgements of distress and anger. For example, individuals show a higher sensitivity to distress cues when viewing own-race faces compared to faces of other racial groups [34]. In addition, pain [35] and sadness are perceived less readily in Black than White individuals [36]. Interestingly, this extends to perceptions of susceptibility to pain as well; pain is perceived as being less easily experienced in Black than White individuals, a finding that could have particular relevance in medical settings [17]. Understanding the impact of medical masks, given they are most likely to be worn in such settings, is of particular importance.

Relative to White individuals, Black individuals tend to be perceived as more aggressive and hostile. For example, facial expressions of anger are significantly more likely to be falsely perceived in Black versus White children [37, 38]. Furthermore, people are more likely to perceive racially ambiguous angry faces as Black compared to racially ambiguous neutral or happy faces [39]. This tendency to differentially assess anger in Black individuals is associated with higher implicit racial prejudice [37, 40].

There is some evidence to suggest that contextual factors like clothing may interact with target race to further influence emotion recognition. The term “hoodie effect” was popularized following the murder of Trayvon Martin in 2014, in which the perpetrator stated Martin looked suspicious due to his choice of clothing [41]. In line with this, evidence exists suggesting that Black individuals were more likely to be assessed as threatening when dressed in a baggy hoodie, a piece of clothing that can obscure parts of the face [42]. These findings show that external factors, such as clothing, may exacerbate the effects of race on emotion recognition and perception.

In light of evidence that mask wearing and target race in isolation can impact emotion recognition performance, and existing concerns that masks and race may interact to exacerbate emotion recognition difficulties, the present study sought to address the collective impact of medical masks on emotional expression recognition as a function of target race. We were particularly interested in the impact these factors have on the recognition of anger and distress due to the important role they play in modulating behaviour during conflict, and their relevance for encounters with police and in healthcare settings. We predicted that participants would be less accurate at recognizing emotions on Black faces, with this effect being exacerbated when masks were present. We predicted that participants would more readily perceive anger, and rate it more intensely, in Black actors than White actors, with masks exacerbating these effects. In contrast, we expected masks to exacerbate a tendency for participants to be less accurate at identifying distress cues (sadness and fear), and to perceive distress cues to be less intense on Black actors than White actors.

## Methods

### Participants

Participants were 148 adults recruited from CloudResearch, a participant-sourcing platform for online research. Eligible participants were between the ages of 18 and 45, and reported no colour vision deficiencies, recent head trauma, neurological conditions (e.g., stroke), current mental health issues, or major medical disorder. Only those who had access to a computer with a Windows operating system were included, as the E-Prime Go task was only compatible with Windows devices. Sixteen participants were unable to complete the experimental task due to technical issues. In total, 134 participants (M_age_ = 32.01, SD = 5.26 years, 93 males and 41 females) successfully completed the study. The breakdown of self-reported participant race is as follows: 15.5% Asian, 7.8% Black, 10.3% Hispanic or Latino, 0.9% Indigenous, 60.3% White, and 5.2% Mixed Race. This experiment was approved by the Health Sciences Research Ethics Board at the University of Western Ontario.

### Procedure

Prospective participants first completed a screening form to determine eligibility for the study. Eligible participants were then sent a Qualtrics survey link containing the Letter of Information and Consent, as well as a demographic questionnaire. Each participant was compensated $10 USD for questionnaire completion. Participants were then provided a link to download the Medical Mask experimental task through the E-Prime Go website. Participants were compensated a further $10 USD for completing the experimental task.

### Medical Mask Task

**Fig 1:**
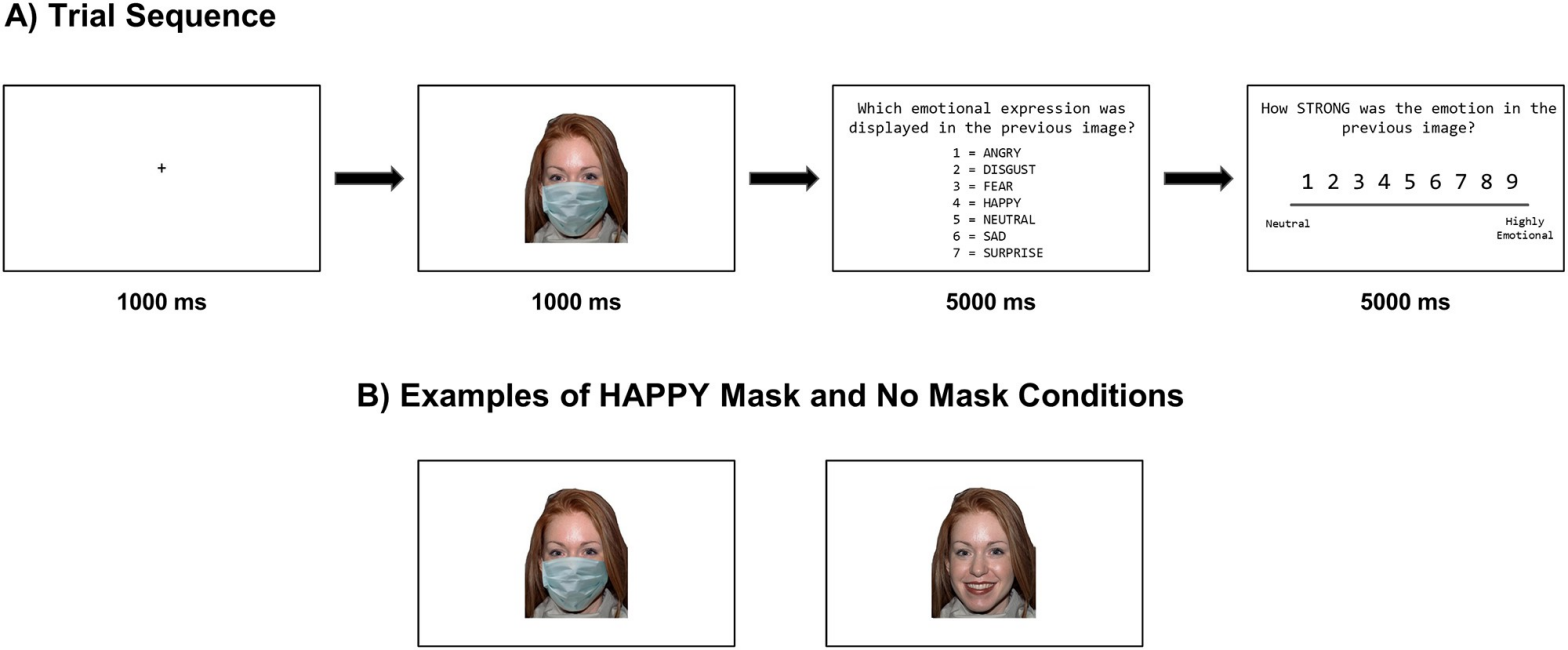
Schematic of the Medical Mask Task (a) Each trial consisted of a fixation cross, and a stimulus (masked or unmasked face) displayed for 1000ms. Participants were then presented with an emotion recognition response screen which listed the seven possible response options corresponding to the six basic emotional expressions plus a neutral expression. This was followed by an emotional intensity rating screen in which participants rated the intensity of the expression on a 9-point likert scale (ranging from neutral to highly emotional). (b) Example of ‘mask’ and ‘no mask’ conditions for the ‘Happy’ emotion condition.

A total of 140 unmasked stimuli were selected from the NimStim Set of Facial Expressions [43] to create the Medical Mask Task in E-Prime Go (see Fig 1). The NimStim Set of Facial expressions is large multiracial database of images of actors depicting eight basic facial expressions designed to test basic emotional expression recognition in typical individuals. Psychometric evaluation lends empirical support for their validity and reliability [43]. For the present study, a subset of stimuli were selected consisted of adult actors depicting one of the six universal facial expressions (anger, fear, sadness, happiness, disgust, surprise) or a neutral expression. For masked stimuli, medical masks were superimposed onto the original images using Procreate for IPAD [44] photo-editing software. Stimuli were balanced across actor race (Black or White), actor gender (male or female) and mask condition (Mask or No Mask). For each trial, a fixation cross appeared at the center of the screen, followed by a stimulus presented for 1000ms. Participants were then asked to indicate which emotional expression was being displayed using keys 1–7 on the keyboard. Option order was randomized across runs. Participants were also asked to rate the intensity of the displayed emotion on a nine-point Likert scale, with 1 representing “neutral” and 9 representing “highly emotional,” and were given 5000ms to respond. The task contains eight blocks with 35 stimuli each for a total of 280 trials distributed equally across conditions. Every block was balanced across actor race, actor gender and emotional expression. Four blocks contained masked stimuli and four contained unmasked stimuli. Block order was pseudo-randomized across participants so that at no time were two consecutive blocks of masked or unmasked stimuli presented.

### Data analysis

Participant accuracy was assessed using the proportion of correct responses for each trial type. Data files from three participants were incomplete and subsequently removed. An additional 13 participants were excluded for performing at below 25% accuracy. The final sample included 116 participants. For the initial analysis, separate 3-way repeated measures analyses of variance (ANOVAs) were performed corresponding to a 2 (Mask: Mask, No Mask) X 2 (Actor Race: Black, White) X 7 (Emotion: Anger, Fear, Sadness, Neutral, Happiness, Disgust, Surprise) for accuracy and intensity scores. Where significant, the nature of interactions were delineated by way of separate 2-way repeated measures ANOVAS corresponding to 2 (Mask) X 2 (Actor Race) repeated-measures design. For all follow-up tests conducted after the omnibus ANOVA (including both follow-up ANOVAs and t-tests), we corrected for multiple comparisons using the Benjamini-Hochberg method [45] calculated separately for accuracy and intensity.

To facilitate a broader understanding of the types of errors, we also conducted a misattribution analysis whereby the tendency to erroneously apply a particular emotional label was examined (e.g., to use the label “anger” for expressions other than anger). To do so, we calculated the mean number of times each emotional label was incorrectly applied, and subjected these data to a 3-way repeated measures ANOVA corresponding to a 2 (Mask) X 2 (Actor Race) X 7 (Emotion) design. These were also subjected to correction according to the Benjamini-Hochberg method [45].

In light of evidence suggesting that there is an in-group advantage for expression recognition [34], exploratory analyses were planned to examine the additional between-subjects factor of Participant Race. For this analysis, a subset of participants identifying as White and Black would form the two groups. However, while 70 White participants took part in the study, 9 participants in the study identified as Black; thus, the analysis was deemed under-powered.

## Results

### Accuracy

**Fig 2:**
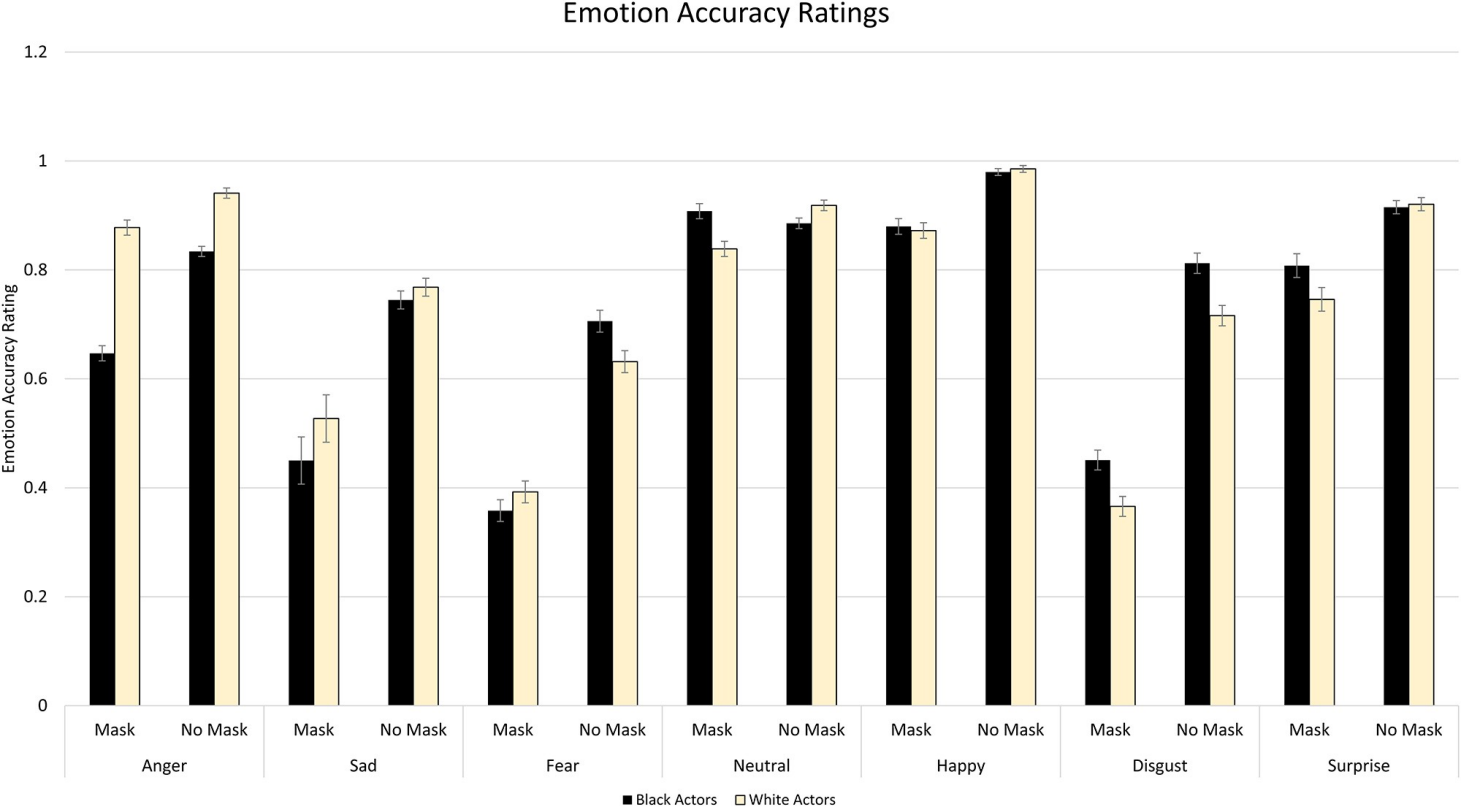
Mean accuracy on the Medical Mask Task. The graph depicts the proportion correct across task conditions. Significant Mask X Actor Race interactions were observed for Anger, Fear, Neutral and Surprise. Error bars reflect within subject’s standard errors.

**Fig 3:**
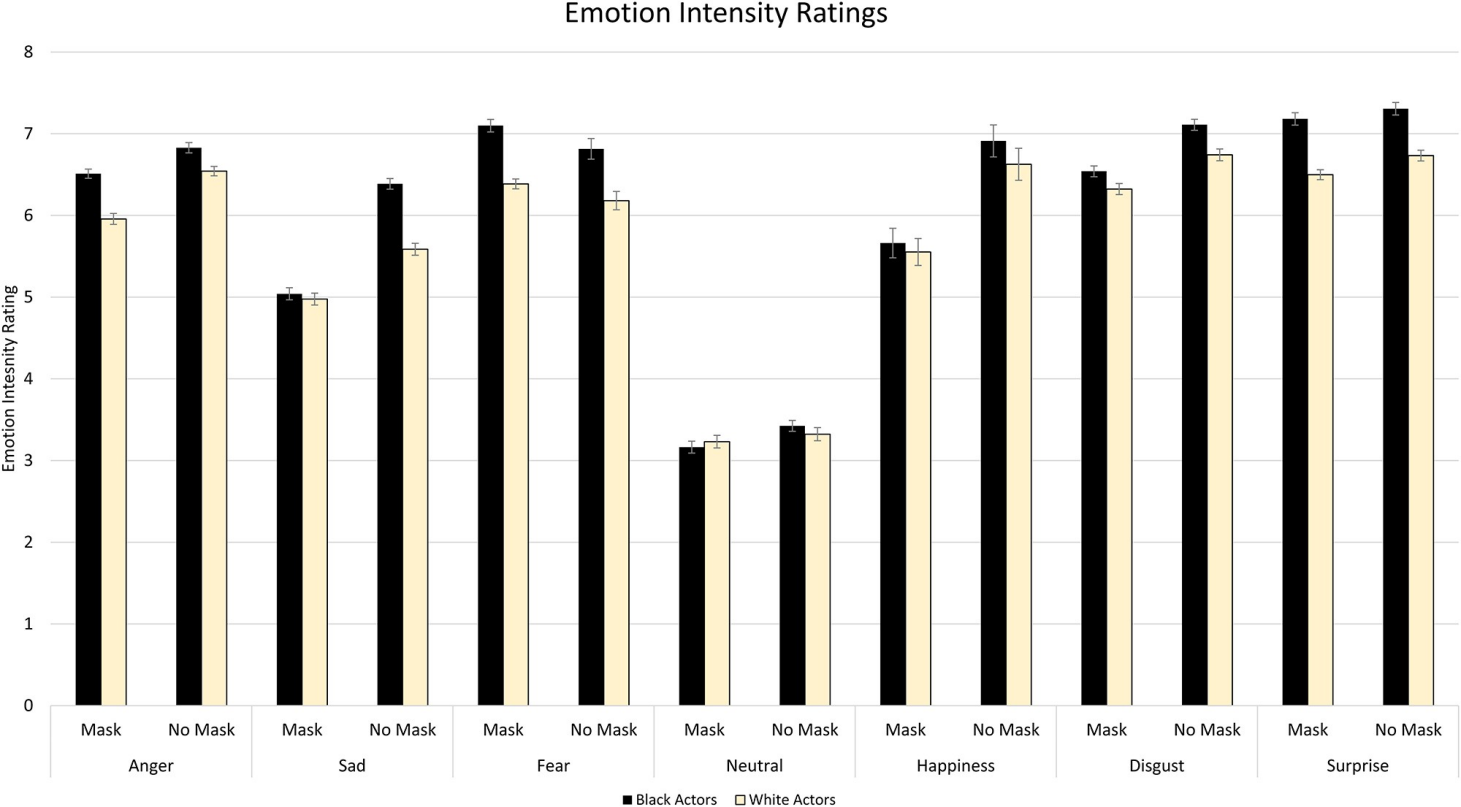
The “cost” of wearing a medical mask on accuracy depicted across emotions. Masks significantly reduced recognition accuracy for all emotional facial expressions. Masks significantly interacted with race only for anger, fear, neutral and surprise (see results regarding the ANOVA). The value of the bars was calculated by subtracting proportion correctly recognized in the unmasked condition from the proportion correctly recognized in the masked condition. Error bars reflect standard error of the mean difference.

To determine the collective impact of actor race and mask wearing on emotion recognition, we first examined the accuracy data across mask status, race and emotion. The full-factorial analysis yielded a main effect of Mask, *F*(1, 115) = 769.58, *p* < .001, η^2^_p_ = .87. As expected, accuracy was significantly lower when masks were present compared to when they were not present. There was no significant effect of Actor Race, *F*(1,115) = 3.548, *p* = .062, η^2^_p_ = .03. A main effect of Emotion was revealed, *F*(6, 690) = 137.24, *p* < .001 η^2^_p_ = .54. Participants were most accurate at identifying Happy expressions, followed by Neutral, Surprise, Anger, Sadness, Disgust and Fear. These effects were qualified by significant interactions. With regard to two way interactions, the Mask X Actor Race interaction was nonsignificant, *F*(1,115) = 2.91, *p* = .091, η^2^_p_ = 0.03. A significant Actor Race X Emotion interaction emerged, *F*(6, 690) = 35.14, *p* < .001, η^2^_p_ = .23, as well as a significant Mask X Emotion interaction, *F*(6,690) = 49.58, *p* < .001, η^2^_p_ = .30. Notably, these effects were qualified by a significant Mask X Actor Race X Emotion interaction, *F*(6, 690) = 10.65, *p* < .001, η^2^_p_ = .09. To better characterize this interaction, seven 2 (Mask) X 2 (Actor Race) ANOVAs were performed (one for each emotion condition). Mean accuracy and the relative “cost” of masks on intensity ratings by condition are depicted in Figs 2 and 3 respectively.

#### Accuracy for anger

For Anger, there was a significant effect of Mask, *F*(1, 115) = 118.45, *p* < .001, η^2^_p_ = .51; participants were less accurate when masks were present. A main effect of Actor Race was also present, *F*(1, 115) = 224.72, *p* < .001, η^2^_p_ = .66, whereby accuracy was greater for White actors than Black actors. This was qualified by a significant Mask X Actor Race interaction, *F*(1, 115) = 46.30, *p* < .001, η^2^_p_ = .29. Accuracy was significantly lower for Black actors than White actors; this effect was more pronounced when masks were present, *t*(115) = -14.30, *p* < .001, compared to when masks were not present, *t*(115) = -8.50, *p* < .001.

#### Accuracy for sadness

For Sadness, there was a significant effect of Mask, *F*(1, 115) = 116.46, *p* < .001, η^2^_p_ = .50, where participants were less accurate when masks were present. A main effect of Actor Race was also evident, *F*(1, 115) = 4.62, *p* = .034, η^2^_p_ = .04, whereby accuracy was greater for White actors than Black actors. There was no significant Mask X Actor Race interaction, *F*(1, 115) = 1.22, *p* = .271, η^2^_p_ = .01.

#### Accuracy for fear

For Fear, there was a significant effect of Mask, *F*(1, 115) = 269.78, *p* < .001, η^2^_p_ = .70; participants were less accurate when masks were present. No significant effect of Actor Race was present, *F*(1, 115) = 2.24, *p* < .137, η^2^_p_ = .02. However, there was a significant Mask X Actor Race interaction, *F*(1, 115) = 22.45 *p* < .001, η^2^_p_ = .16; accuracy was greater for Black actors than White actors when masks were not present, *t*(115) = 4.06, *p* < .001, but greater in White actors than Black actors when masks were present, though this effect did not survive correction, *t*(115) = -2.05, *n*.*s*.

#### Accuracy for neutral

For Neutral, there was a significant effect of Mask, *F*(1, 115) = 7.03, *p* = .009, η^2^_p_ = .06, whereby participants were less accurate when masks were present. No significant main effect of Actor Race was present, *F*(1, 115) = 3.50, *p* = .064, η^2^_p_ = .03. However, a significant Mask X Actor Race interaction was found, *F*(1, 115) = 33.77, *p* < .001, η^2^_p_ = .23. Accuracy was greater for White actors than Black actors when masks were not present, *t*(115) = -2.67, *p* = .001, but greater for Black actors than for White actors when masks were present, *t*(115) = 5.00, *p* < .001.

#### Accuracy for happiness

For Happiness, there was a significant effect of Mask, *F*(1, 115) = 42.49, *p* < .001, η^2^_p_ = .27, whereby participants were less accurate when masks were present. No significant effect of Actor Race was found, *F*(1, 115) = 0.02, *p* = .903, η^2^_*p*_ < .001, and there was no significant Mask X Actor Race interaction, *F*(1, 115) = 0.71, *p* = .401, η^2^_p_ = .006.

#### Accuracy for disgust

For Disgust, there was a significant effect of Mask, *F*(1, 115) = 423.72, *p* < .001, η^2^_p_ = .79, where participants were less accurate when masks were present. A main effect of Actor Race was also present, *F*(1, 115) = 41.37, *p* < .001, η^2^_p_ = .27; accuracy was greater for Black actors compared to White actors. There was no significant Mask X Actor Race interaction, *F*(1, 115) = 0.22, *p* = .643, η^2^_p_ = .002.

#### Accuracy for surprise

For Surprise, there was a significant effect of Mask, *F*(1, 115) = 76.16, *p* < .001, η^2^_p_ = .40, whereby participants were less accurate when masks were present. A main effect of Actor Race was also present *F*(1, 115) = 8.11, *p* = .005, η^2^_p_ = .07, whereby accuracy was greater for Black actors than White actors. This was qualified by a significant Mask X Actor Race interaction, *F*(1, 115) = 11.68, *p* < .001, η^2^_p_ = .09. Accuracy was greater for Black actors than White actors when masks were present, *t*(115) = 3.72, *p* < .001, but no significant difference was found when masks were not present, *t*(115) = - 0.51, *p* = .611.

### Intensity

**Fig 4:**
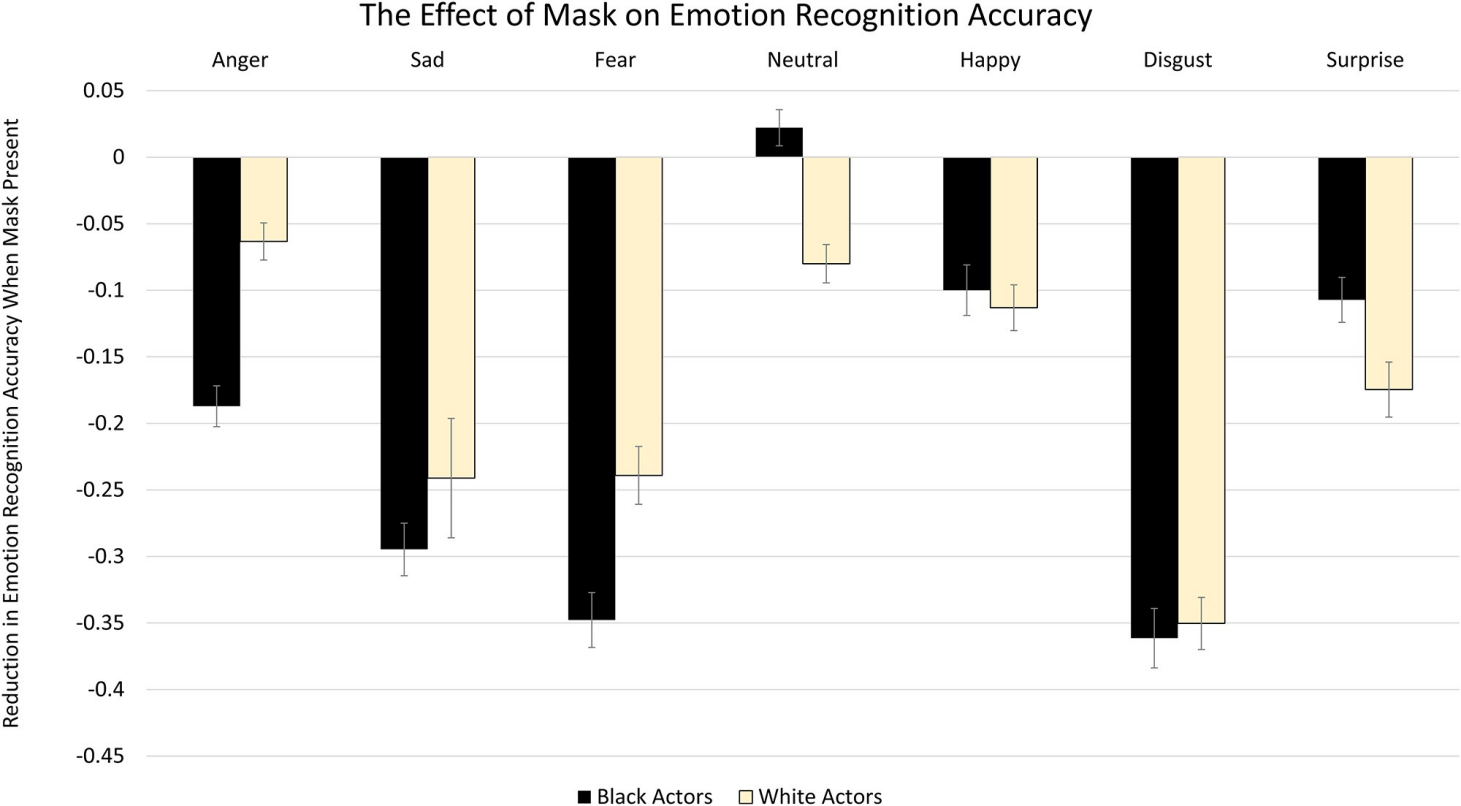
Mean intensity ratings for the Medical Mask Task. Numbers reflect the average rated intensity of the displayed emotion on a nine-point Likert scale, with 1 representing “neutral” and 9 representing “highly emotional.” Significant Mask X Actor Race interactions were observed for anger, sadness and happiness. Error bars reflect within subject’s standard errors.

**Fig 5:**
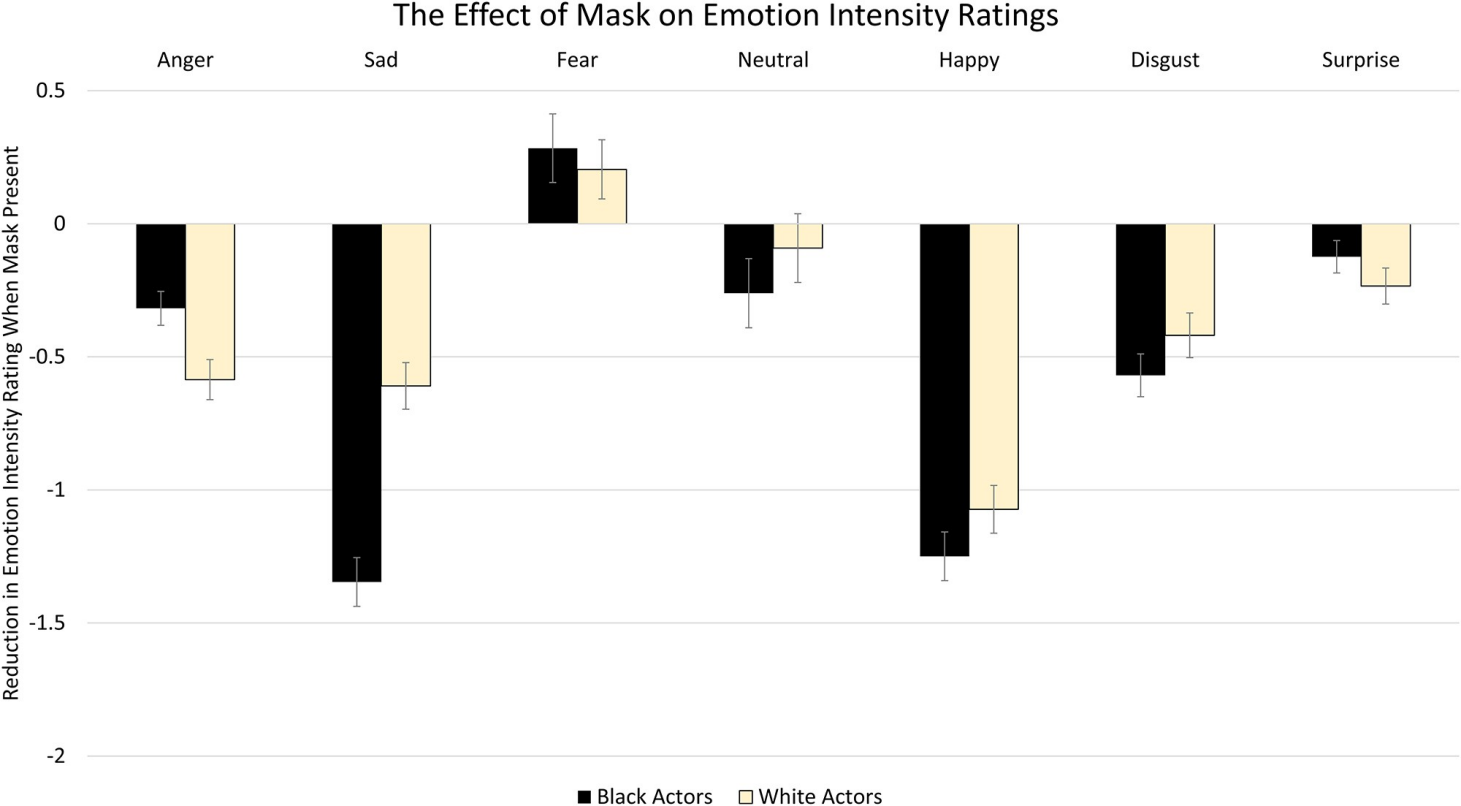
The “cost” of wearing a medical mask on intensity ratings depicted across emotions. Medical masks significantly reduced intensity ratings across emotional facial expressions for all expressions except for neutral (no effect overall) and fear (masked fear was rated as more intense than unmasked fear). Masks significantly interacted with race only for the emotions anger, sadness and happiness (see results regarding the ANOVA). The value of the bars was calculated by subtracting the rated intensity during the unmasked condition from the rated intensity of the same expressions during the masked condition. Error bars reflect standard error of the mean difference.

To determine the collective impact of actor race and mask wearing on emotion intensity judgements, next we examined the intensity ratings as a function of mask status, race and emotion. The full-factorial analysis revealed a main effect of Mask, *F*(1, 115) = 176.12, *p* < .001, η^2^_p_ = .61, whereby masks decreased the perceived intensity of emotions. A main effect of Actor Race was also found, *F*(1, 115) = 244.00, *p* < .001, η^2^_p_ = .68, whereby the overall perceived intensity of emotion was higher for Black actors in comparison to White actors. Additionally, a main effect of Emotion emerged, *F*(6, 690) = 198.70, *p* < .001, η^2^_p_ = .63. Participants rated expressions of Surprise as most intense, followed by Disgust, Fear, Anger, Happiness, and Sadness. As expected, neutral expressions were rated as least intense. These effects were qualified by a significant Mask X Actor Race X Emotion interaction, *F*(6, 690) = 16.38, *p* < .001, η^2^_p_ = .13. To better characterize this interaction, seven 2 X 2 ANOVAs were performed examining the interaction between Mask and Actor Race conditions for each emotion condition. Mean intensity ratings and the impact of masks on intensity ratings by condition are depicted in Figs 4 and 5 respectively.

#### Intensity for anger

For Anger, there was a significant effect of Mask, *F*(1, 115) = 59.14, *p* < .001, η^2^_p_ = .34; participants perceived angry expressions as less intense when a mask was present. A significant effect of Actor Race was also discovered, *F*(1, 115) = 82.15, *p* <. 001, η^2^_p_ = .42, whereby anger was perceived as more intense on Black actors relative to White actors. There was a significant Mask X Actor Race interaction, *F*(1, 115) = 12.61, *p* < .001, η^2^_p_ = .10. The difference in intensity ratings for Black versus White actors was more pronounced when masks were present, *t*(115) = 9.37, *p* < .001, than when masks were not present, *t*(115) = 4.74, *p* < .001.

#### Intensity for sadness

For Sadness, there was a significant effect of Mask, *F*(1, 115) = 157.89, *p* < .001, η^2^_p_ = .58, whereby participants perceived sad expressions as less intense when a mask was present. A significant effect of Actor Race was also discovered, *F*(1, 115) = 78.66, *p* <. 001, η^2^_p_ = .41, whereby sadness was perceived as more intense on Black actors relative to White actors. This was qualified by a significant Mask X Actor Race interaction, *F*(1, 115) = 68.37, *p* < .001, η^2^_p_ = .37. While sadness was perceived as more intense on Black actors than on White actors when masks were not present, *t*(115) = 12.77, *p* < .001, there was no significant difference when masks were present, *t*(115) = 0.93, p = .353.

#### Intensity for fear

For Fear, there was a significant effect of Mask, *F*(1, 115) = 4.59, *p* = .03, η^2^_p_ = .04; participants perceived fearful expressions as more intense when a mask was present. A significant effect of Actor Race was also discovered, *F*(1, 115) = 139.01, *p* <. 001, η^2^_p_ = .55, whereby fear was perceived as more intense on Black actors relative to White actors. There was no significant Mask X Actor Race interaction, *F*(1, 115) = 1.06, *p* = .306, η^2^_p_ = .009.

#### Intensity for neutral

For Neutral, there was no significant effect of Mask, *F*(1, 115) = 2.09, *p* = .151, η^2^_p_ = .02, nor was there a significant effect of Actor Race, *F*(1, 115) = 0.22, *p* = .640, η^2^_p_ = .002. No significant Mask X Actor Race interaction was found, *F*(1, 115) = 3.74, *p* = .055, η^2^_p_ = .03.

#### Intensity for happiness

For Happiness, there was a significant effect of Mask, *F*(1, 115) = 189.46, *p* < .001, η^2^_p_ = .62, whereby participants perceived happy expressions as less intense when a mask was present. A significant effect of Actor Race was also discovered, *F*(1, 115) = 16.52, *p* < .001, η^2^_p_ = .13, whereby happiness was perceived as more intense on Black actors relative to White actors. This was qualified by a significant Mask X Actor Race interaction, *F*(1, 115) = 7.10, *p* = .009, η^2^_p_ = .06. Significantly greater intensity ratings for Black relative to White actors were observed when masks were not present, *t*(115) = 4.74, *p* < .001, but not when masks were present, *t*(115) = 1.91, *p* = .059.

#### Intensity for disgust

For Disgust, there was a significant effect of Mask, *F*(1, 115) = 50.25, *p* < .001, η^2^_p_ = .30, whereby participants perceived expressions of disgust as less intense when a mask was present. A significant effect of Actor Race was also discovered, *F*(1, 115) = 47.23, *p* <. 001, η^2^_p_ = .29, whereby disgust was perceived as more intense on Black actors relative to White actors. There was no significant Mask X Actor Race interaction, *F*(1, 115) = 3.00, *p* = .086, η^2^_p_ = .03.

#### Intensity for surprise

For Surprise, there was a significant effect of Mask, *F*(1, 115) = 12.14, *p* < .001, η^2^_p_ = .10, whereby participants perceived surprised expressions as less intense when a mask was present. A significant effect of Actor Race was also discovered, *F*(1, 115) = 189.47, *p* <. 001, η^2^_p_ = .62, whereby surprise was perceived as more intense on Black actors relative to White actors. No significant Mask X Actor Race interaction was found, *F*(1, 115) = 2.02, *p* = .158, η^2^_p_ = .02.

### Misattribution analyses

We next sought to investigate the frequency of false positive ratings as a function of Mask, Actor Race, and Emotion (see Table 1 for a breakdown of emotion-specific error rates). To do so, a 2 (Mask) X 2 (Actor Race) X 7 (Emotion) ANOVA was performed. The analysis revealed a main effect of Mask, *F*(1, 115) = 905.554, *p* < .001, η^2^_p_ = .89, whereby masks increased the prevalence of false positive attributions. There was no significant effect of Actor Race, *F*(1, 115) = 3.18, *p* < .077, η^2^_p_ = .027. A main effect of Emotion also emerged, *F*(6,690) = 77.593, *p* < .001, η^2^_p_ = .40; false positive ratings were most prevalent for expressions of Surprise followed by Anger, Fear, Disgust, Neutral, Sad, and Happy. These effects were qualified by a significant Mask X Actor Race X Emotion interaction, *F*(6,690) = 4.611, *p* < .001, η^2^_p_ = .039. To better characterize this interaction, seven 2 (Mask) X 2 (Actor Race) ANOVAs were performed examining the interaction of these factors for each of the seven emotion conditions.

**Table 1:** Emotion-specific error rates by condition.

			Facial Expression Identified															
			Anger		Sad		Fear		Neutral		Happy		Disgust		Surprise		SUM	
			Black Actor	White Actor	Black Actor	White Actor	Black Actor	White Actor	Black Actor	White Actor	Black Actor	White Actor	Black Actor	White Actor	Black Actor	White Actor	Black Actor	White Actor
**Facial Expression Presented**	Anger	Masked	64.01	87.07	3.02	1.19	7.54	1.08	4.63	3.66	0.54	0.65	17.03	6.14	3.23	0.22	100	100
		Unmasked	82.76	93.75	1.72	0.97	1.29	0.43	0.64	0.54	0.00	0.54	12.07	3.66	1.51	0.11	100	100
	Sad	Masked	13.36	8.62	44.61	48.38	9.59	16.16	16.92	16.81	0.97	1.51	12.93	5.93	1.62	2.59	100	100
		Unmasked	1.51	2.48	73.38	76.61	8.94	9.59	1.51	2.91	0.32	0.22	12.72	7.11	1.62	1.08	100	100
	Fear	Masked	0.65	2.05	0.43	4.85	35.99	40.09	0.43	1.72	0.32	0.65	1.08	2.37	61.10	48.28	100	100
		Unmasked	0.65	3.56	0.54	3.99	70.69	63.69	1.08	0.97	0.22	0.11	3.34	7.22	23.49	20.47	100	100
	Neutral	Masked	1.62	5.06	4.42	3.34	0.43	2.05	90.09	83.51	1.40	1.72	1.19	1.40	0.86	2.91	100	100
		Unmasked	3.34	2.69	6.90	2.05	0.11	0.97	87.18	91.81	1.40	1.19	0.75	0.97	0.32	0.32	100	100
	Happy	Masked	1.72	2.80	0.86	0.75	0.65	1.19	7.97	6.03	87.50	87.28	0.97	1.29	0.32	0.65	100	100
		Unmasked	0.32	0.22	0.11	0.11	0.97	0.65	0.54	0.75	97.84	97.52	0.11	0.32	0.11	0.43	100	100
	Disgust	Masked	44.18	46.01	4.96	8.73	1.94	3.99	2.05	1.40	1.61	2.37	44.50	36.42	0.75	1.08	100	100
		Unmasked	11.31	16.81	4.42	7.65	2.48	2.48	0.11	0.54	0.11	0.65	81.25	71.34	0.32	0.54	100	100
	Surprise	Masked	0.65	0.65	0.11	0.97	18.32	19.83	0.43	4.2	0.43	0.43	0.43	1.19	79.63	72.74	100	100
		Unmasked	0	0.11	0.75	0.32	7.22	5.82	0.43	0.65	0.11	1.19	0.86	0.65	90.63	91.27	100	100
	False Positive Ratings	Masked	577	605	128	184	357	411	301	314	49	68	312	170	630	517	2354	2269
		Unmasked	159	240	134	140	195	185	40	59	20	36	277	185	254	213	1079	1058

*Note*: The numerical values presented for each of the 7 facial expressions identified (columns) reflect the average percentage of all responses allocated for each of those 7 emotions presented (rows). The final column “SUM” merely illustrates that each value adds up to 100%. For the “False Positive Ratings” row, the values reflect the total number of times a particular emotion label erroneously applied for each emotion, and the total number of errors is given in the final “SUM” column.

#### Anger misattributions

For false perceptions of Anger, there was a significant effect of Mask, *F*(1,115) = 339.99, *p* < .001, η^2^_p_ = .747, such that participants were more likely to falsely perceive anger when masks were present. A significant effect of Actor Race was also discovered, *F*(1, 115) = 12.20, *p* < .001, η^2^_p_ = .096, whereby participants were more likely to misattribute anger to White actors than Black actors. There was no Mask X Actor Race interaction, *F*(1, 115) = 2.589, *p* = .110, η^2^_p_ = .022.

#### Sad misattributions

For false perceptions of Sadness, there was no significant effect of Mask, *F*(1, 115) = 1.39, *p* = 0.241, η^2^_p_ = .012. However, a significant effect of Actor Race was discovered, *F*(1, 115) = 6.61, *p* = .011, η^2^_p_ = .054, whereby participants were more likely to misattribute sadness to White actors. This was qualified by a significant Mask X Actor Race interaction, *F*(1, 115) = 5.25, *p* = .024, η^2^_p_ = .044. Participants were significantly more likely to misattribute sadness to White actors versus Black actors when masks were present, *t*(115) = -3.36, *p* = .001. However, this effect did not reach significance when masks were not present, *t*(115) = -0.378, *p* = .706.

#### Fear misattributions

For erroneous perceptions of Fear, there was a significant effect of Mask, *F*(1, 115) = 84.41, *p* < .001, η^2^_p_ = .423, whereby participants wrongly perceived fear more frequently when masks were present. However, there was no significant effect of Actor Race, *F*(1, 115) = 2.21, *p* = .140, η^2^_p_ = .019. These effect were qualified by a significant Mask X Actor Race interaction, *F*(1, 115) = 6.17, *p* = .014, η^2^_p_ = .051. While participants were significantly more likely to misattribute fear to White actors relative to Black actors in the masked condition, *t*(115) = -2.37, *p* = .019, but no difference was observed when masks were not present, *t*(115) = 0.631, *p* = 0.529.

#### Neutral misattributions

For falsely perceiving Neutral, there was a significant effect of Mask, *F*(1, 115) = 135.178, *p* < .001, η^2^_p_ = .540, such that participants were more likely to misperceive neutral expressions when masks were present. There was no significant effect of Actor Race, *F*(1, 115) = 1.62, *p* = .206, η^2^_p_ = .014, nor was there a significant Mask X Actor Race interaction, *F*(1, 115) = 0.06, *p* = .810, η^2^_p_ = .001.

#### Happy misattributions

For false perceptions of Happiness, there was a significant effect of Mask, *F*(1, 115) = 12.51, *p* < .001, η^2^_p_ = .098, whereby participants misperceived emotions as happy more frequently when masks were present. A significant effect of Actor Race was also discovered, *F*(1, 115) = 10.43, *p* = .002, η^2^_p_ = .083, whereby happiness was falsely attributed to White actors more than to Black actors. There was no significant Mask X Actor Race interaction, *F*(1, 115) = 0.049, *p* = .826, η^2^_p_ = .000.

#### Disgust misattributions

For false perceptions of Disgust, there was no significant effect of Mask, *F*(1, 115) = 0.354, *p* = .553, η^2^_p_ = .003. However, a significant effect of Actor Race was discovered, *F*(1, 115) = 56.55, *p* < .001, η^2^_p_ = .330, whereby participants were more likely to misattribute disgust to Black actors. There was no significant Mask X Actor Race interaction, *F*(1, 115) = 3.86, *p* = .052, η^2^_p_ = .032.

#### Surprise misattributions

For Surprise, there was a significant effect of Mask, *F*(1, 115) = 385.94, *p* < .001, η^2^_p_ = .770, whereby participants were more likely to misperceive surprise when masks were present. A significant effect of Actor Race was also discovered, *F*(1, 115) = 39.88, *p* <. 001, η^2^_p_ = .257, whereby surprise was more likely to be misattributed to Black actors. This was qualified by a significant Mask X Actor Race interaction, *F*(1, 115) = 11.752, *p* < .001, η^2^_p_ = .093. While participants were significantly more likely to misattribute surprise to Black actors than White actors in the unmasked condition, *t*(115) = 2.55, *p* = .012, this effect was exacerbated when masks were present, *t*(115) = 7.02, *p* < .001.

## Discussion

In the present study, we determined the collective impact of medical masks on emotional expression recognition as a function of actor race. For each of the six basic emotional facial expressions, medical masks were associated with significantly more overall emotional expression recognition errors. Masks also significantly reduced perceived intensity of emotions, except for fear, wherein masks were associated with increased intensity ratings. Overall, the effects associated with race varied depending on the emotion and appearance of masks. Whereas for anger and sadness recognition accuracy was higher for White relative to Black actors, the opposite pattern was observed for disgust. Mask wearing significantly interacted with actor race to impact facial expression processing for only a subset of emotions. With respect to accuracy, masks tended to exacerbate actor-race related recognition differences for anger and surprise, but attenuated these differences for fear. For emotional intensity ratings, masks tended to increase race-related differences for judgements of anger, but decrease these differences for sad and happy expressions. Overall, our results suggest that the interaction between actor race and mask wearing status with respect to emotional expression judgements is complex, dependent on the emotion, and varying in direction and degree. We consider the implications of these results particularly with regard to emotionally charged social exchanges, such as that which can occur during conflict, healthcare, and policing.

### Anger and distress

We were particularly interested in interactions involving race and mask wearing for the processing of emotions associated with anger and distress (sadness/fear) given the crucial role they are thought to play in modulating the social behaviours of observers [22, 29–31]. We observed that anger expression recognition was significantly worse for Black relative to White actors. Despite being less accurately recognized, anger was rated as significantly more intense in Black than White actors. Medical masks exacerbated the size of this race-related effect, both in terms of reducing accuracy and increasing intensity, indicating an asymmetrical impact of masks for Black and White actors. This is a particularly important consideration given that interpreting anger in others is associated with efforts to de-escalate conflict through initiating appeasement gestures [24] and other contextual modification of observers’ behaviours [21–23]. If present in naturalistic settings, reduced anger recognition coupled with enhanced perceived intensity of that expression in Black versus White individuals could disrupt appropriate and proportional responding in emotionally charged situations, including healthcare and policing.

Like anger, distress cues such as sadness and fear are thought to modulate social behaviour; however, whereas anger is thought to increase avoidance behaviours, distress cues are thought to engender sympathy and approach-related helping behaviours [22, 46] while reducing the likelihood of aggression [47]. With regard to distress cues in Black relative to White actors, we expected that participants would make more recognition errors, have lower emotional intensity ratings, and that these effects would be exacerbated by mask-wearing. However, the pattern of results observed were more complex. Participants were less accurate at recognizing sadness in Black versus White participants, as was predicted, but this pattern was not exacerbated by masks. Conversely, fear was better recognized in Black than White actors for unmasked, but not masked faces. Also contrary to predictions, fear was rated more intensely in Black versus White actors (irrespective of mask-wearing); in addition, sad unmasked faces were perceived as more intense in Black than White actors, but this effect was absent when masks were worn. It is difficult to predict the motivational impact of such effects should they appear in naturalistic settings. Abnormalities in recognition accuracy for distress cues, even bidirectional ones, may have implications for contexts in which distress cues can influence outcomes. It is possible that increased perception of distress may lead to more helping behaviours as has been observed in some settings [32]. Alternatively, such perceptions can complicate interactions in other ways. For example, trustworthiness judgements have important implications for social decision making and outcomes in a variety of domains including politics, business, and the law [48]. We observed increased fear appraisal in Black versus White actors (characterized by increased recognition for unmasked faces only, but increased intensity ratings regardless of mask status). Evidence suggests that people with fearful facial expressions are rated as less trustworthy by student police officers than those with positive facial expressions [49]. Therefore, an enhanced appraisal of fear could have implications for judgements and decision making that take trustworthiness into account during police encounters. In the context of healthcare, there is considerable evidence that pain is underestimated and undertreated in Black patients [50, 51]. Although the factors contributing to this issue are likely multifaceted, abnormalities in the perception of distress have been found to correlate with such disparities in medical treatment [52]. Our finding that sadness was less likely to be recognized in Black than White actors, and that masks reduce the recognition accuracy and perceived intensity of sadness for both White and Black actors, is important to consider in the context of healthcare, where some decisions are impacted by perceptions of distress.

### Other emotions

For the emotions other than anger and distress, actor race and mask status interacted for neutral and surprised expressions with respect to accuracy, and only for happiness with respect to perceptions of intensity. Neutral expressions were better recognized in White actors than Black actors for unmasked faces, but the opposite was true when masks were introduced. For surprise, significant differences in recognition accuracy by actor race only emerged when masks were present, characterized by a disproportionate reduction in accuracy for White relative to Black actors. The significance of this finding is less clear as surprise is elicited by unanticipated events, and the same surprised expression can be interpreted by viewers as either positive or negative depending on the context [53]. Given this, it may be useful for future studies to include ratings of valence as an additional outcome measure.

There were no effects of actor race observed for happy expression recognition accuracy; however, masks eliminated the tendency to rate happiness as more intense in Black than White actors. Disgust was more accurately recognized in Black versus White actors irrespective of mask status. The potential interpersonal impact that this pattern of results might have in naturalistic settings is unclear. Disgust is thought to signal the presence of potentially harmful pathogens in the environment [54–56]. The concept of moral disgust has also arisen, which is thought to emerge in response to behaviours that violate established social norms such as incest or murder [55, 57]. Perhaps most importantly given the current results, similarities between facial expressions of disgust and those of contempt have been noted [54]; thus, asymmetries in the sensitivity across races and mask contexts could potentially be interpreted as hostility, and thereby contribute to variance in the nature of social interactions.

### Confusion matrices

To better understand the impact of medical masks and race on emotion recognition, we were interested not only in the errors when emotional expressions were present (false negatives), but also the misattribution of a label when the emotion was absent (false positives). In light of recent findings involving prospective teachers’ misattributions of anger and hostility in Black individuals [37, 38], we had predicted that participants would be more likely to misattribute anger for Black versus White actors. Furthermore, we predicted that masks would exacerbate this tendency. Contrary to expectations, misattributions of anger were greater for White actors regardless of mask status; thus, participants were less likely to perceive anger in Black actors whether it was there or not, and whether or not a mask was worn.

It should be noted that the current study differs from those of Halberstadt et al., [37, 38] in that it did not target prospective teachers, it involved only adult rather than child actors, and included only prototypical rather than more nuanced emotional expressions. Many studies that report racialized biases in anger tend to focus on implicit associations with threat and hostility [15, 16], or identification amongst a smaller subset of emotions rather than against the backdrop of six or more basic emotional expressions [40, 58]. Indeed, other studies examining basic emotional expression processing have found that White individuals tend to show higher error rates for emotional expression recognition in Black actors more generally [59]. Although numerous studies concerning in-group advantages for emotion recognition have been conducted [34], comparatively few have explicitly examined basic emotion expression recognition in Black versus White actors. The implications of the pattern of results we observed were complex. Of note, a greater tendency to identify anger when it is not present, as was seen here in White versus Black actors, could have arisen from numerous factors and/or contribute to numerous outcomes. Anger is associated with increased perceptions of dominance, and people tend to be more sensitive to recognizing anger in those who are considered dominant [60]. A tendency to perceive anger when it is not present could complicate social interactions in multiple ways. For example, when perceived in individuals who would be considered dominant, it could lead to higher rates of appeasement and conciliatory behaviours; conversely, when perceived in individuals who are considered lower in the dominance hierarchy, such a bias could increase the risk for conflict [60, 61]. In contrast, failing to perceive anger when it is present (as was observed more often for Black actors), could reduce the likelihood of appeasement or conciliatory gestures, and therefore increase the risk of inappropriate responses and escalation. Although we found that masks had a disproportionate impact on misattributions of anger in White versus Black actors, it is important to note that masks significantly increased the tendency to see anger in both (i.e., people were several times more likely to misperceive anger in masked faces than non-masked faces). This finding is worthy of further investigation given the potential adverse consequences of misperceiving threat in a community setting.

The confusion matrix also revealed some interactions with actor race and/or mask status. Although there was no effect of race on false positives for sadness and fear for unmasked faces, the introduction of masks were associated with significantly higher likelihood of mistaking other emotions for sadness and fear when actors were White relative to Black. For distress cues, participants were significantly more likely to misidentify sadness and fear to White actors than Black actors, but only when masks were present.

### Limitations and future directions

We were most interested in the effects of mask wearing on emotional expression processing as a function of race and emotion. As a consequence, we chose stimuli for which the normative supplementary data presented in Tottenham et al. [43] indicated that the Black and White actors selected were reasonably matched in terms of accuracy. Nevertheless, we did observe some potentially important effects of race that were independent of mask use, and it would be important to replicate these results in a more diverse set of stimuli. The study would also benefit from expanding the stimuli to include other racialized groups. This is also true for the sample under investigation, which predominantly consisted of people who identified as White individuals. It would be of interest to examine these effects in a much larger and culturally diverse sample, allowing for an analysis of interactions with in-group and out-group as a function of membership. Finally, we used only stereotypical, static emotional facial expressions. It would be useful to include more ambiguous stimuli (e.g., by varying the intensity), and include dynamic facial expressions to better mimic how emotions are expressed in naturalistic settings.

### Conclusions

Given our reliance on non-verbal cues such as facial expressions to guide our interactions with others, it is important to elucidate the potential impact of medical masks on social situations as they become more common in public settings (e.g., healthcare settings). The present study highlighted the collective impact of medical masks on emotional expression recognition as a function of actor race. Results suggest that the interaction between actor race and mask wearing status with respect to emotional expression judgements is complex, varying across emotion, in both direction and degree. Masks tended to promote race-related recognition differences for anger (where recognition advantages for White versus Black actor were increased by masks) and surprise (where recognition was better for Black versus White actors only for masked stimuli). However, masks attenuated race-related differences for fear; accuracy was greater for Back versus White actors only when masks were not present. For emotional intensity ratings, masks tended to increase race-related differences for judgements of anger (greater intensity ratings for Black versus White actors). However, differences associated with actor race were attenuated for both sad and happy expressions where significantly increased intensity ratings for Black versus White actors were observed only when masks were not present. Understanding the impact of medical masks on the fundamental building blocks of social communication is central to identifying and reducing potential biases in the context of emotionally charged social contexts, such as in healthcare, during conflict, and in encounters with law enforcement.

## Supporting information

S1 Table Normative information for the stimuli selected for the current study. *Note*. Normative Kappa values and proportion correct are shown for the validated NimSTIM images selected for the present study (see Tottenham et al., 2009 supplementary tables for details). *p < 0.*05*.
(DOCX)
