# Supplementary material for: Masking our emotions: Emotion recognition and perceived intensity differ by race and use of medical masks
Source: PLoS One. 2023 Jun 7;18(6):e0284108. doi: 10.1371/journal.pone.0284108 (PMC10246819; doi:10.1371/journal.pone.0284108)
Supplement: S1 Table — Note. Normative Kappa values and proportion correct are shown for the validated NimSTIM images selected for the present study (see Tottenham et al., 2009 supplementary tables for details). *p < 0.05. (DOCX) [file pone.0284108.s001.docx]

**Supplementary Table S1.** *Normative information for the stimuli selected for the current study*

|  |  | **Facial Expression** | | | | | | | | | | | | | |
| --- | --- | --- | --- | --- | --- | --- | --- | --- | --- | --- | --- | --- | --- | --- | --- |
|  |  | Anger | | Sad | | Fear | | Neutral | | Happy | | Disgust | | Surprise | |
|  |  | Black Actors | White Actors | Black Actors | White Actors | Black Actors | White Actors | Black Actors | White Actors | Black Actors | White Actors | Black Actors | White Actors | Black Actors | White Actors |
| Kappa | Mean | 0.85 | 0.80 | 0.73 | 0.78 | 0.70 | 0.64 | 0.86 | 0.84 | 0.99* | 0.95* | 0.78 | 0.69 | 0.69 | 0.73 |
|  | Range | 0.25 | 0.29 | 0.21 | 0.24 | 0.37 | 0.31 | 0.28 | 0.29 | 0.03 | 0.09 | 0.26 | 0.52 | 0.41 | 0.26 |
|  | Minimum | 0.72 | 0.63 | 0.60 | 0.69 | 0.49 | 0.53 | 0.68 | 0.67 | 0.97 | 0.90 | 0.63 | 0.40 | 0.50 | 0.55 |
|  | Maximum | 0.97 | 0.92 | 0.81 | 0.93 | 0.86 | 0.84 | 0.96 | 0.96 | 1.00 | 0.99 | 0.89 | 0.92 | 0.91 | 0.81 |
| Percentage Correct | Mean | 0.88 | 0.93 | 0.84 | 0.79 | 0.76 | 0.65 | 0.89 | 0.93 | 0.99 | 0.99 | 0.83 | 0.66 | 0.82 | 0.85 |
|  | Range | 0.32 | 0.26 | 0.35 | 0.38 | 0.34 | 0.38 | 0.36 | 0.13 | 0.01 | 0.01 | 0.16 | 0.66 | 0.24 | 0.19 |
|  | Minimum | 0.66 | 0.73 | 0.63 | 0.60 | 0.59 | 0.46 | 0.63 | 0.85 | 0.99 | 0.99 | 0.74 | 0.31 | 0.71 | 0.75 |
|  | Maximum | 0.98 | 0.99 | 0.98 | 0.98 | 0.93 | 0.84 | 0.99 | 0.98 | 1.00 | 1.00 | 0.90 | 0.97 | 0.95 | 0.94 |

*Note.* Normative Kappa values and proportion correct are shown for the validated NimSTIM images selected for the present study (see Tottenham et al., 2009 supplementary tables for details).

**p < 0.05*
